# 

**Published:** 1886-10-02

**Authors:** 


					CONTENTS.
Notices -
To our Readers
Discharged.
By George Fleming.
CHAPTER I -
4
Hospital Administration.?
Mr. Sturge and the Alco-
hol Question
Words of Consolation
r lowers, Ferns, and Ward Decorations
Notes and News
The Story of the Hospitals.
By One of the Public
Hospital Worthies.
Dr. Jas. Goodchild Wakley
The Editor's Letter Box.-
Offices' Food, a-d the
Regulation of Meals
Poetry-
The Blind
Difficult Cases
General Inquiries
Help at Hand, Brother - M
Prize Competitions
1 he Children s Corner
The Hospital Charity Pox
The Out-Patients' Diary -
PUBLISHED AT
THE OFFICE OF "THE HOSPITAL," 30 & 32, SARDINIA STREET, LINCOLN'S INN FIELDS.
To he had of all Booksellers and Newsagents in the United Kingdom, and at all the
Railway Bookstalls of Messrs. W. H. Smith & Son.

				

